# Supplementary material for: T cell assays differentiate clinical and subclinical SARS-CoV-2 infections from cross-reactive antiviral responses
Source: Nat Commun. 2021 Apr 6;12:2055. doi: 10.1038/s41467-021-21856-3 (PMC8024333; doi:10.1038/s41467-021-21856-3)
Supplement: Supplementary file 1 — Supplementary Information [file 41467_2021_21856_MOESM1_ESM.pdf]

# Supplementary figure 1

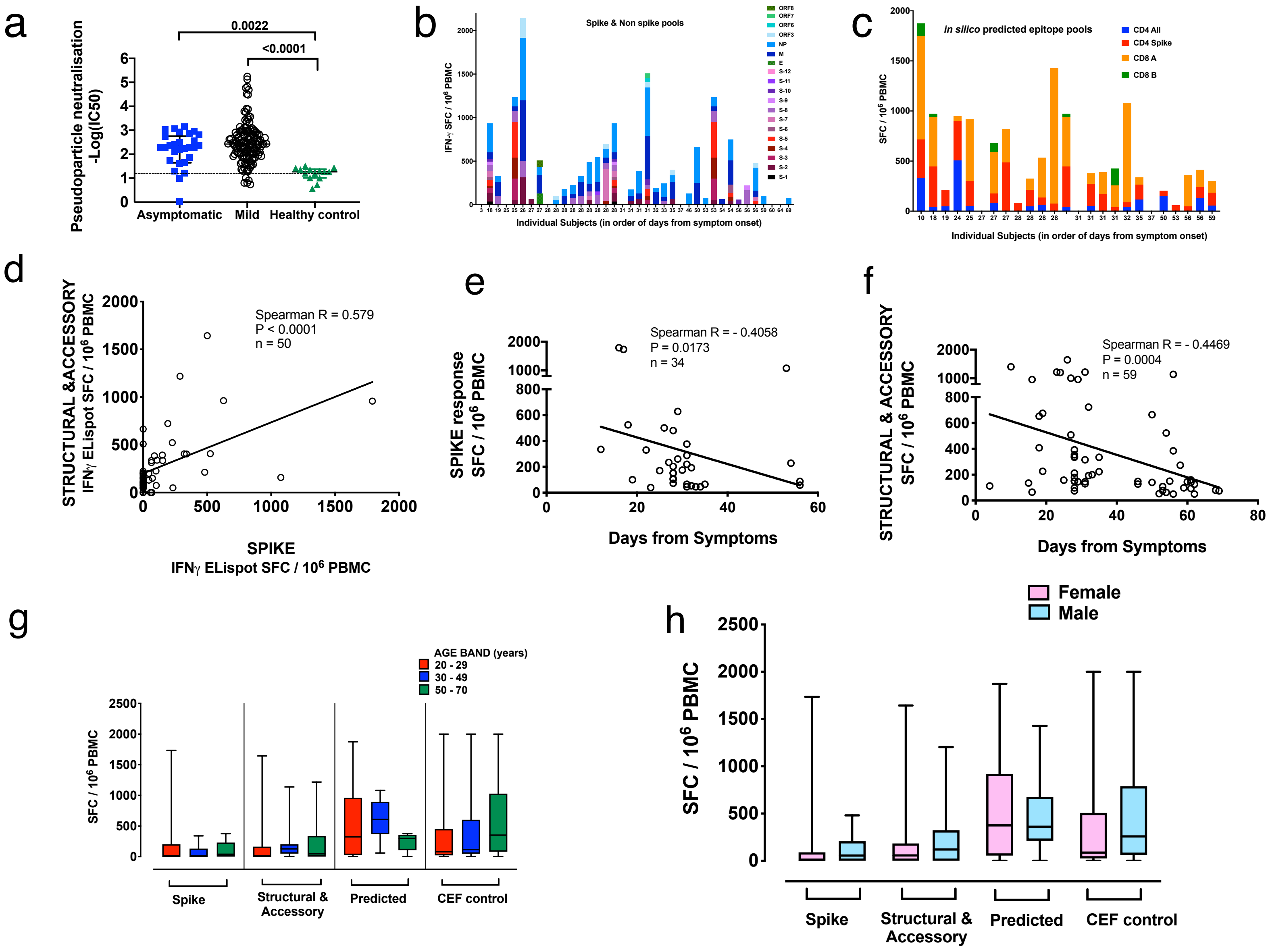


**Detailed SARS-CoV-2 specific Immune Response.**

a) Neutralising antibody responses in a pseudoparticle assay in 30 asymptomatic and 146 mildly symptomatic healthcare workers (HCWs) with PCR-confirmed SARS-CoV-2 infection, and 14 seronegative controls. *Ex vivo* IFN-γ ELISpot showing the magnitude and breadth of effector T cell responses to b) SARS-CoV-2 spike peptide pools and M, NP, and accessory proteins ORF 3, ORF6, ORF7 and ORF8 (n=38) and c) *in silico* predicted pools[10] in individual HCWs convalescent with mildly symptomatic SARS-CoV-2 infection (n=25). X axis shows number of days from onset of symptoms (not to scale), with blank columns representing zero response in the individual tested at that time-point. d) Correlation between summed *ex vivo* IFN-γ ELISpot responses to spike protein and to structural and accessory proteins (n=50). Correlation between days from symptom onset and summed *ex vivo* IFN-γ ELISpot responses to e) spike protein (n=34) and f) structural and accessory proteins (n=59). *Ex vivo* IFN-γ ELISpot responses to summed SARS-CoV-2 peptide pools spanning spike, accessory and structural proteins (E, M, NP, ORF 3, ORF6, ORF7 and ORF8), *in silico* predicted pools[10] and the CEF T cell control panel by g) age band in years (20-29 years n=41, 30-49 years n=43, 50-70years n=27), and by h) sex (female n=74, male n=37). SFC/$10^6$ PBMC = spot forming cells per million peripheral blood mononuclear cells, with background subtracted. Correlation was performed via Spearman's rank correlation coefficient and comparison of three groups for age by Kruskal-Wallis one-way ANOVA and two groups for sex by Mann-Whitney U test. ns = not significant, * = <0.05, ** = <0.01, *** = < 0.001 and **** = <0.0001.

# Supplementary figure 2

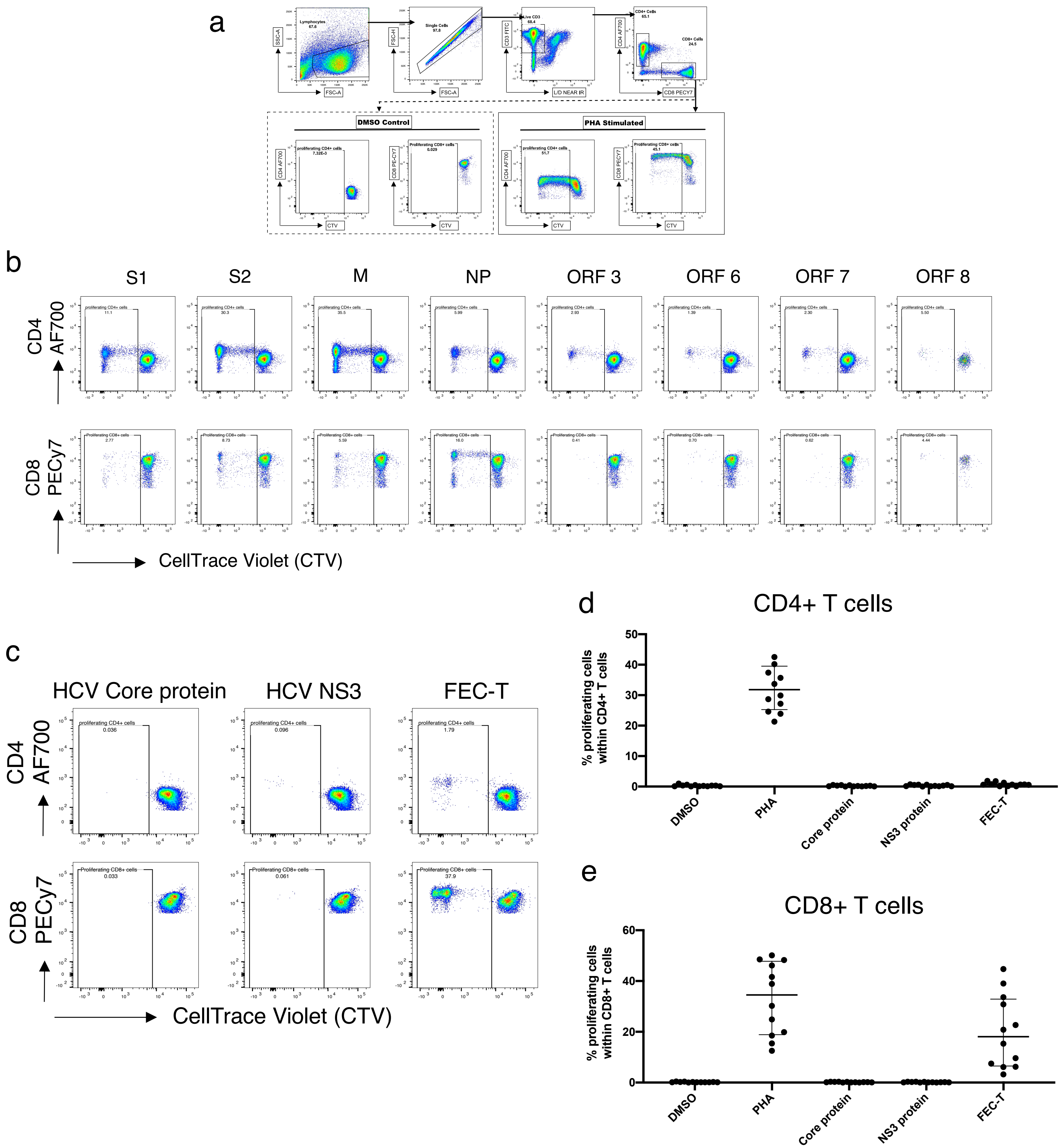


**Gating strategy and representative plots for CTV assay.**

a) Gating strategy used for the identification of proliferating CD4+ and CD8+ T cells as analysed in figures 3(a, b, c,), 6(a, b, c, d, f, g), 7(b, c, d, e, f, g), supplementary figure 2(d, e), supplementary figure 3 and supplementary figure 6. b) Representative plots for gating peptide pool specific proliferative responses. c) Representative plots for validation of specificity of proliferation assay using HCV seronegative samples d) quantification of HCV and FEC-T specific protein response in CD4+ and e) CD8+ T cells. PHA is used as positive control. All data have been background subtracted. Each datapoint represents a single volunteer and plots show median with error bars indicating +/- IQR. n=11 volunteers.

# Supplementary figure 3

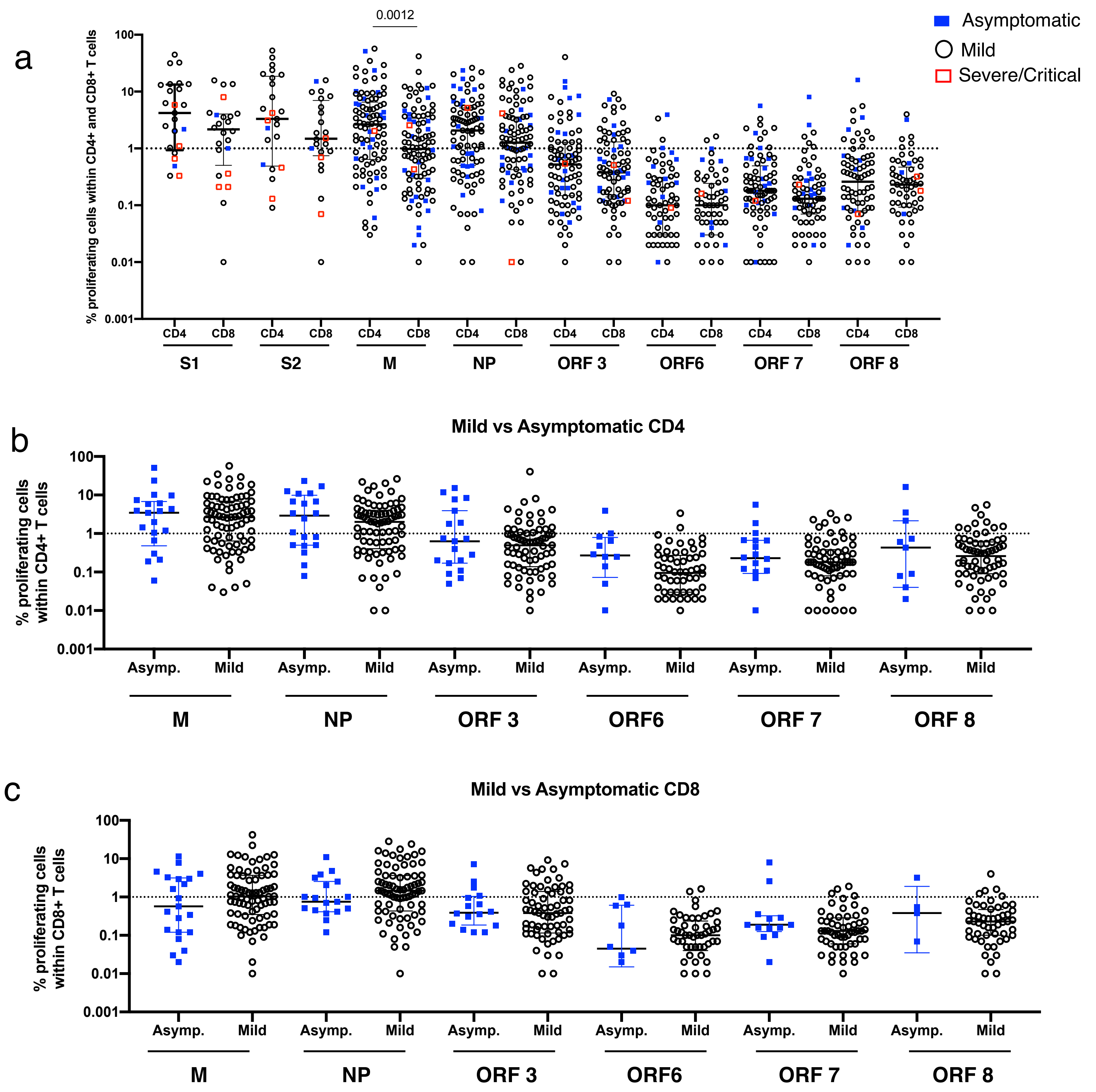


**Comparative analysis of the magnitude of proliferative responses.**

a) CD4+ and CD8+ T cells. b) mild but symptomatic and asymptomatic convalescent HCWs within the CD4+ T cell population and c) mild but symptomatic and asymptomatic convalescent HCWs within the CD8+ T cell population. Number of volunteers: asymptomatic = 23, mild = 88, severe = 4, critical = 2. Each datapoint represents a single volunteer and plots show median with error bars indicating +/- IQR. Comparison of two groups was done by Mann-Whitney U test. Where indicated, ns = not significant, * = <0.05, ** = <0,01, *** = < 0.001 and **** = <0.0001

# Supplementary figure 4

a

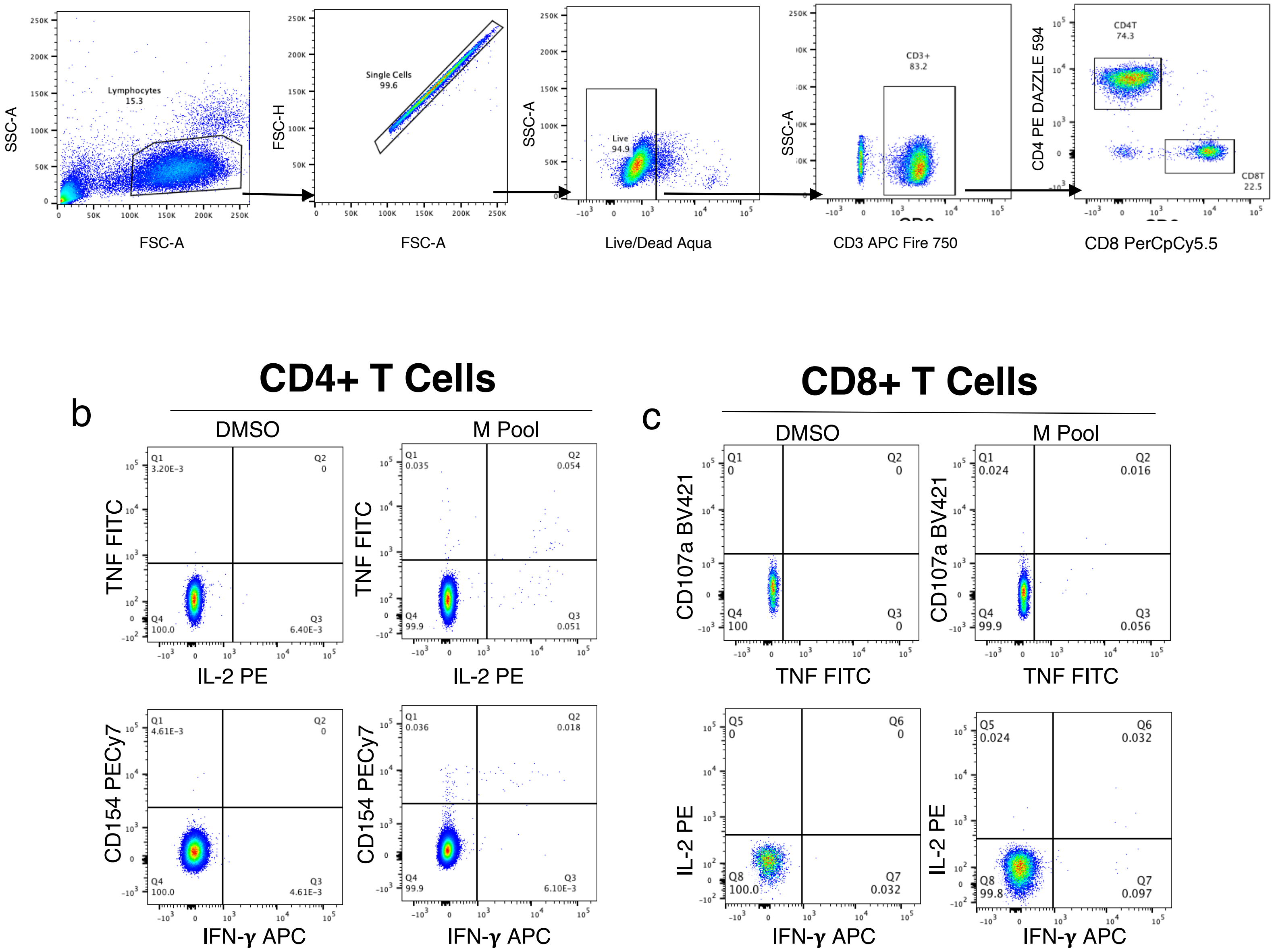


**Representative ICS plots**

PBMC were stimulated with 2ug/mL of the indicated peptide pool or DMSO control. The gating strategy for CD4+ and CD8+ T cells is shown in (a). Representative plots for gated CD4+ T cells are shown in (b) and gated CD8+ T cells in (c). Gating strategy was used for analysis of figures 4 and supplementary figure 5

# Supplementary figure 5

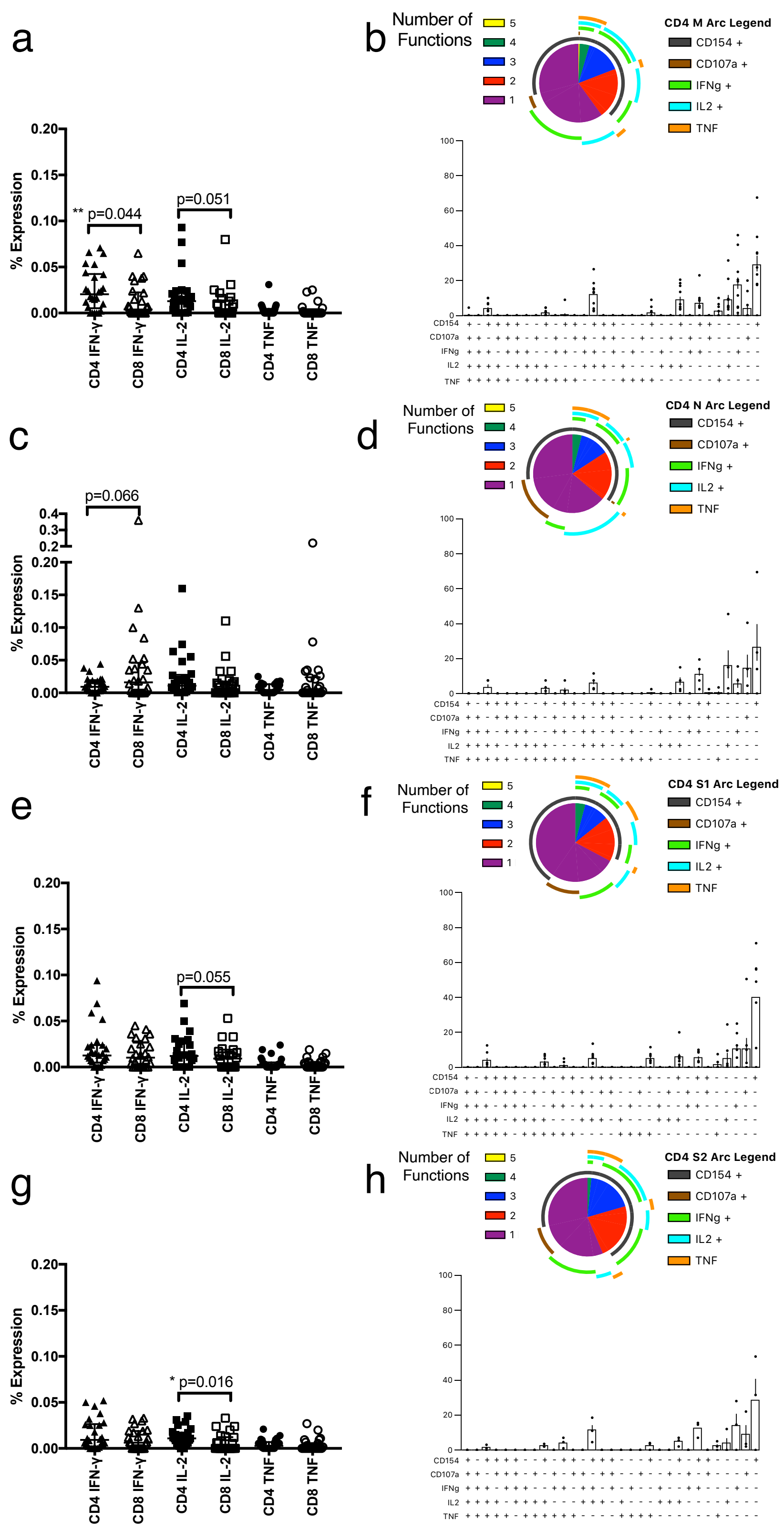


**ICS responses in CD4+ and CD8+ T cells for Spike Pools.**

ICS was performed as in Figure 4 on n=26 individuals PCR+ for SARS-CoV-2. Expression levels of IFN-γ, IL-2 and TNF in CD4+ and CD8+ T cells are shown for the peptide pools M in (a), NP in (c), S1 in (e), and S2 in (g). Bars represent median +/- IQR. Statistics were performed using two-tailed Wilcoxon matched-pairs signed rank test between each cytokine in CD4+ vs CD8+ T cells. Polyfunctionality was then assessed as in Figure 4. Multiple cytokine expression is shown for CD4+ T cells for M pools in (b) n=10, NP pools in (d) n=4, S1 pools in (f) n=6, and S2 pools in (h) n=3.

# Supplementary figure 6

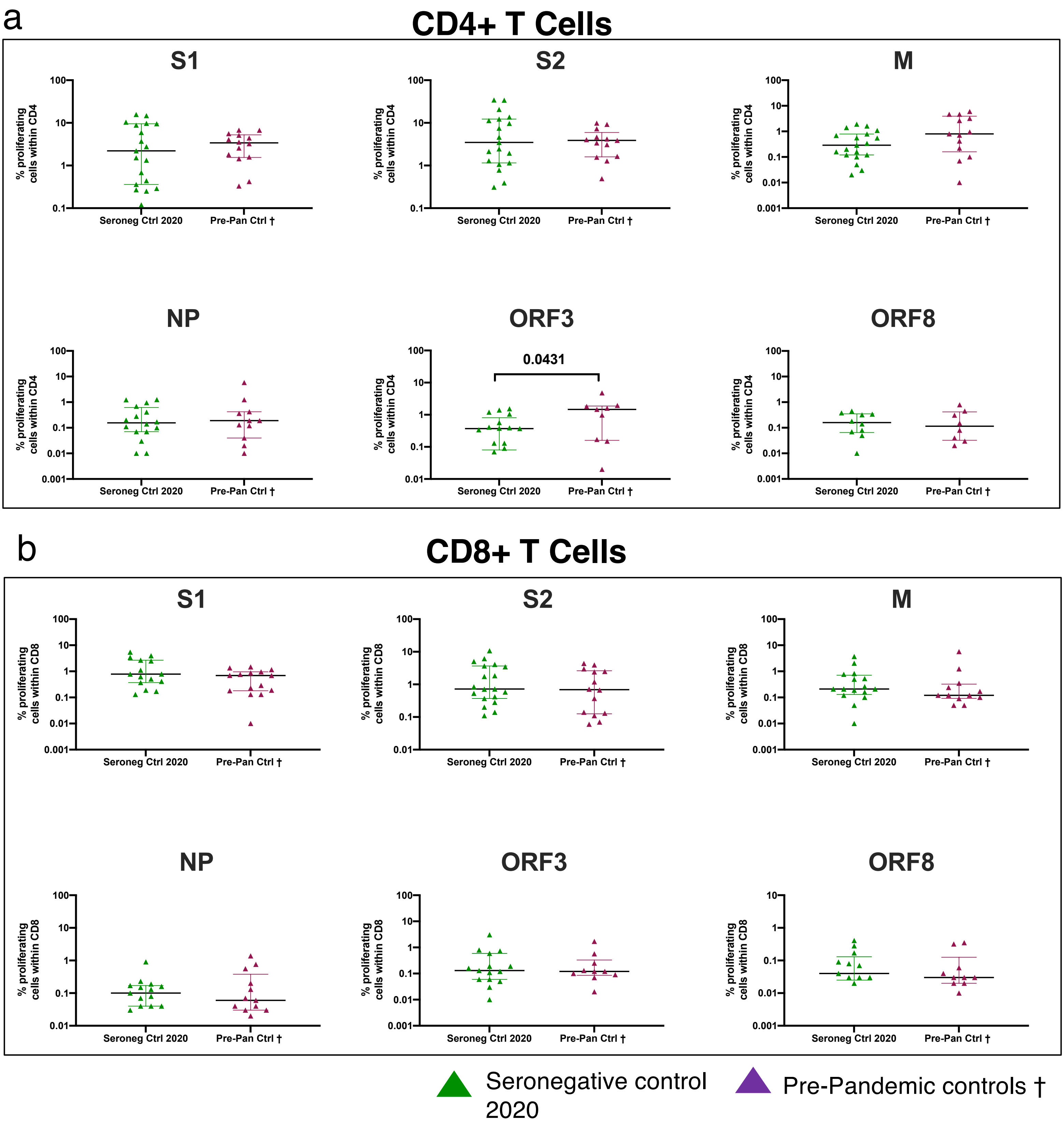


**Proliferative responses in seronegative samples from 2020 and pre-pandemic.**

Comparative analysis of the magnitude of proliferative responses in SARS-COV-2 seronegative controls from 2020 (n=22) analysed from fresh PBMCs and cryopreserved pre-pandemic seronegative controls (n=15) in a) CD4+ and b) CD8+ T cells. Each datapoint represents a single volunteer and plots show median with error bars indicating +/- IQR. Comparison of two groups was done by two-tailed Mann-Whitney U test. Where indicated, ns = not significant, * = <0.05, ** = <0,01, *** = < 0.001 and **** = <0.0001

# Supplementary figure 7

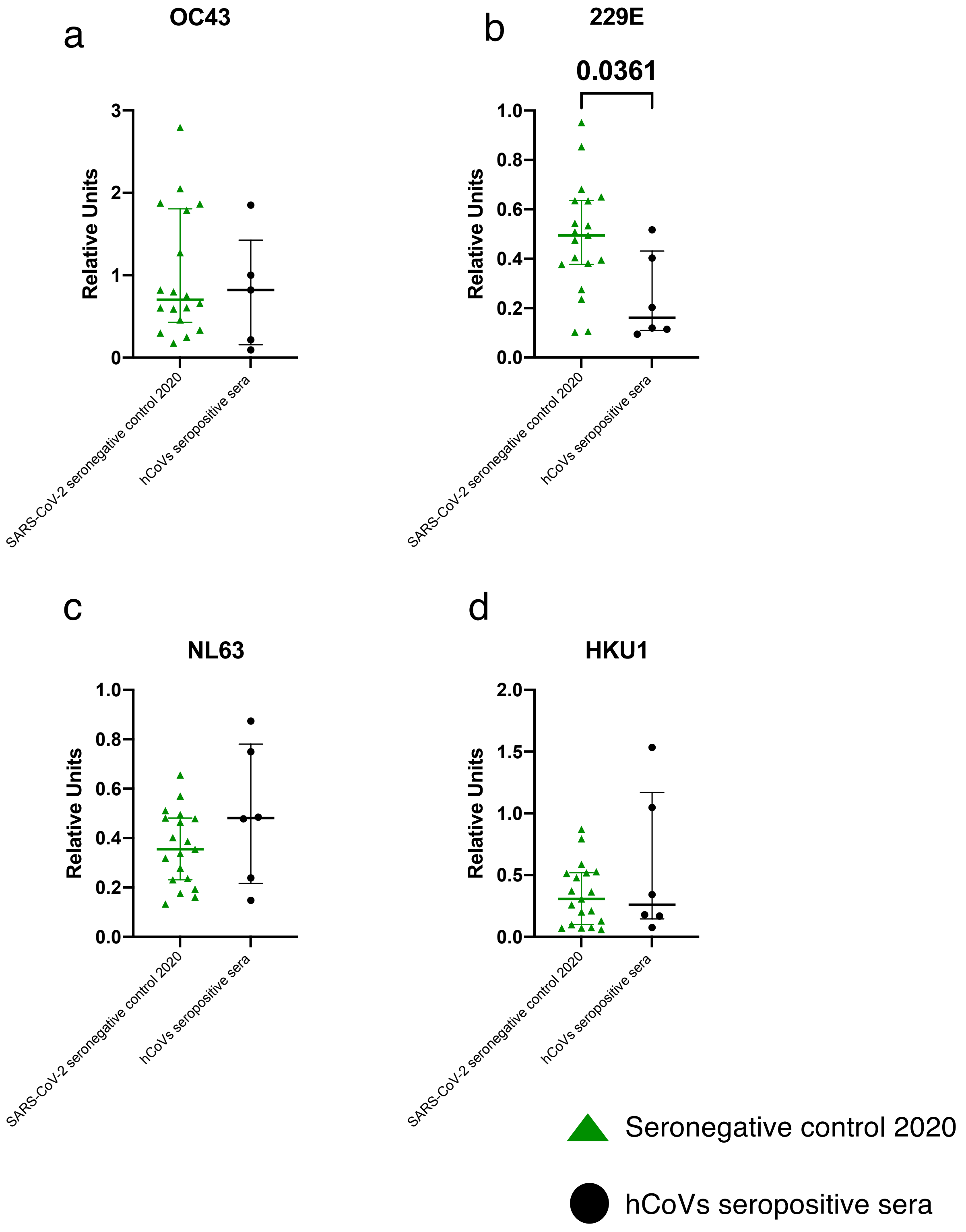


**Serologic responses to circulating HCoVs in seronegative individuals.**

The sera from 19 SARS-CoV-2 seronegative individuals used in the T cell assays (with a pool of 6 seropositives for comparison) were assessed for reactivity against spike proteins derived from the four major circulating coronaviruses – a) OC43, b) 229E, c) NL63 and d) HKU1) as indicated. Each datapoint represents a single volunteer and plots show median with error bars indicating +/- IQR. Comparison of two groups was done by two-tailed Mann-Whitney U test. Where indicated, ns = not significant, * = <0.05, ** = <0,01, *** = < 0.001 and **** = <0.0001

# Supplementary figure 8

a

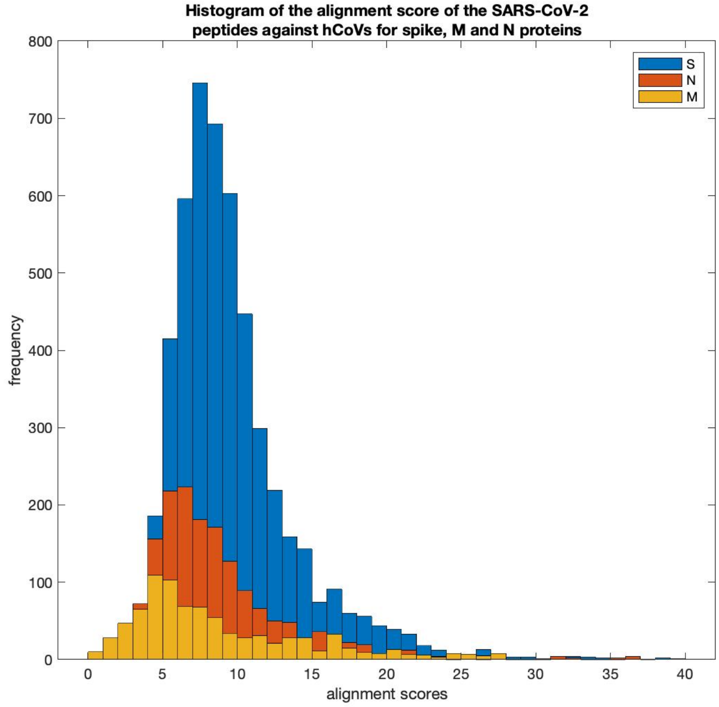


b

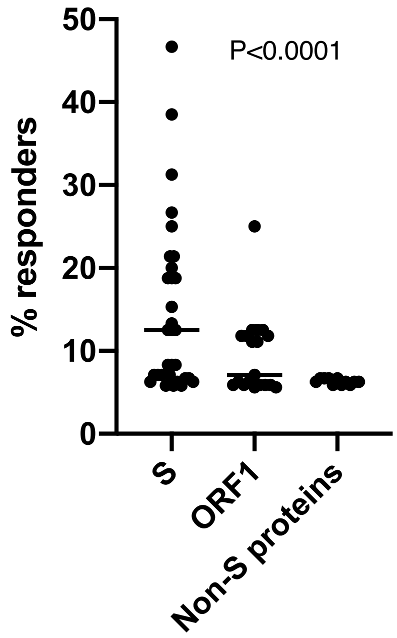


c

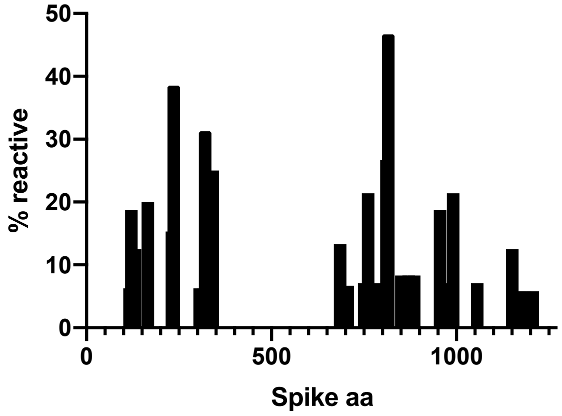


**Analyses of crossreactivity of S1 and S2 vs other antigens from SARS-CoV-2**

a) *Cross reactivity of S vs M and NP at a peptide level.* The SARS-CoV-2 spike, M and NP proteins were divided into 9-mer peptides (with overlap of 8 amino acids). These peptides were then aligned against the homologues proteins of the four species of human coronaviruses (OC43, NL63, 229E and HKU1,) to find the equivalent peptide. The alignment score was recorded for each peptide (higher values indicated higher conservation between SARS-CoV-2 peptide and the equivalent peptide in the HCoVs). The median alignment score for each of spike, NP and M proteins was 8.5, 7.5 and 6.5 respectively which indicates that on average SARS-CoV-2 spike protein peptides are more conserved relative to the HCoVs than M and NP proteins (Mann-Whitney U-test p-values: S vs. NP < $2.2 \times 10^{-16}$, S vs. M < $2.2 \times 10^{-16}$). Additionally focusing on the top 5% most homologous peptides (alignment score > 18.5) across the three proteins, we observed that 61%, 20% and 19% of the peptides were in S, NP and M proteins respectively. The histogram of these alignment scores is plotted for each of the spike, M and NP proteins. b) *Crossreactivity of S vs non-S peptides derived from published datasets.* Samples from the SARS-CoV-2 seronegative population from Matues et al[21] were tested against pools totalling 61 peptides selected for maximal crossreactivity derived from S and non-S regions of SARS-CoV-2. b) The % responders to these peptides is shown, indicating more consistent reactivity against S compared to non-S (ORF1 or other structural proteins) p<0.0001 by two-tailed Welch's ANOVA. c) data from b) plotted across S to demonstrate responses are against both S1 and S2 regions of spike.

# Supplementary Table 1

| Group | N | Sex F/M (%) | Age - median (IQR) | Time from PCR - median (IQR) | Time from symptoms - median, (IQR) |
|---|---|---|---|---|---|
| Asymptomatic | 33 | 26/7 (79/21%) | 36 (27-47) | 6 (5-28) | n/a |
| Mild | 126 | 92/34 (73/27%) | 34.5 (27-48) | 29 (24-54) | 34.5 (27-57) |
| Severe & Critical | 9 | 3/6 (33/67%) | 52 (41-69) | 18 (9-24) | 23 (13-29) |
| TOTAL PCR-confirmed SARS-CoV-2 infection | 168 | | | | |
| Healthy controls: Contemporaneous (Oxford) | 30 | 13/17 (43/57%) | 37 (33-47) | n/a | n/a |
| Healthy controls: Contemporaneous (Sheffield) | 13 | 7/6 (54/46%) | 39 (32-46) | n/a | n/a |
| Healthy controls: Pre-Pandemic (Oxford) * | 19 | 6/4 (60/40%) | 25 (21-38) | n/a | n/a |
| Healthy controls: Pre-Pandemic (Liverpool) | 48 | 35/13 (73/27%) | 26 (22-38.5) | n/a | n/a |
| PCR Negative Inpatients | 9 | 4/5 (44/56%) | 74 (47-79) | n/a | n/a |
| TOTAL Unexposed controls | 119 | | | | |
| Highly Exposed - PCR & Antibody negative | 10 | 3/7 (30/70%) | 31 (25-34) | n/a | n/a |
| P value (ANOVA) | | 0.002 | <0.0001 | <0.0001 | 0.03 |

*missing demographic data for 9 samples

Clinical Information for Patients used in the Study

# Supplementary Table 2

| Antigen stimulants | Spike | | | Structural & Accessory Proteins | | | Predicted Epitopes | | |
|---|---|---|---|---|---|---|---|---|---|
| Group | N | Median (IQR) SFC/$10^6$ | Res-ponder (%) | N | Median (IQR) SFC/$10^6$ | Res-ponder (%) | N | Median (IQR) SFC/$10^6$ | Res-ponder (%) |
| Asymptomatic SARS-CoV-2 infection | 22 | 0 (0-0) | 4/22 (18%) | 23 | 43 (0-148) | 12/23 (52%) | 2 | 28 (0-55) | 1/2 (50%) |
| Mild SARS-CoV-2 infection | 46 | 49 (0-207) | 27/46 (59%) | 73 | 108 (0-325) | 47/73 (64%) | 24 | 205 (0-381) | 21/24 (88%) |
| Severe / critical SARS-CoV-2 infection | 7 | 97 (0-919) | 3/7 (43%) | 7 | 113 (0-223) | 6/7 (86%) | 3 | 130 (0-1780) | 2/3 (67%) |
| Healthy seronegative controls | 22 | 0 (0-0) | 0/22 (0%) | 23 | 0 (0-0) | 1/23 (4%) | 5 | 0 (0-43) | 1/5 (20%) |
| Hospitalised PCR negative | 7 | 0 (0-0) | 0/7 (0%) | 6 | 0 (0-0) | 0/6 (0%) | 5 | 0 (0-0) | 0/5 (0%) |

*Ex vivo* interferon-gamma ELISpot Responses by Group

# Supplementary Table 3

| | Dunn's multiple comparisons test | Summary of P value | Adjusted P Value |
|---|---|---|---|
| SPIKE | Control vs. Asymptomatic | ns | >0.9999 |
| | Control vs. Mild HCW | **** | <0.0001 |
| | Control vs. Severe/Critical | ns | 0.0777 |
| | Control vs. Negative inpatient | ns | >0.9999 |
| | Asymptomatic vs. Mild HCW | * | 0.0276 |
| | Asymptomatic vs. Severe/Critical | ns | 0.6870 |
| | Asymptomatic vs. Negative inpatient | ns | >0.9999 |
| | Mild HCW vs. Severe/Critical | ns | >0.9999 |
| | Mild HCW vs. Negative inpatient | * | 0.0394 |
| | Severe/Critical vs. Negative inpatient | ns | 0.2678 |
| STRUCTURAL & ACCESSORY | Control vs. Asymptomatic | ns | 0.0984 |
| | Control vs. Mild HCW | **** | <0.0001 |
| | Control vs. Severe/Critical | *** | 0.0003 |
| | Control vs. Negative inpatient | ns | >0.9999 |
| | Asymptomatic vs. Mild HCW | ns | 0.8993 |
| | Asymptomatic vs. Severe/Critical | ns | 0.1755 |
| | Asymptomatic vs. Negative inpatient | ns | 0.7317 |
| | Mild HCW vs. Severe/Critical | ns | >0.9999 |
| | Mild HCW vs. Negative inpatient | * | 0.0386 |
| | Severe/Critical vs. Negative inpatient | ** | 0.0090 |
| PREDICTED | Control vs. Asymptomatic | ns | >0.9999 |
| | Control vs. Mild HCW | * | 0.0410 |
| | Control vs. Severe/Critical | ns | >0.9999 |
| | Control vs. Negative inpatient | ns | >0.9999 |
| | Asymptomatic vs. Mild HCW | ns | 0.8620 |
| | Asymptomatic vs. Severe/Critical | ns | >0.9999 |
| | Asymptomatic vs. Negative inpatient | ns | >0.9999 |
| | Mild HCW vs. Severe/Critical | ns | >0.9999 |
| | Mild HCW vs. Negative inpatient | * | 0.0114 |
| | Severe/Critical vs. Negative inpatient | ns | 0.8517 |
| CEF POSITIVE CONTROL | Control vs. Asymptomatic | ns | >0.9999 |
| | Control vs. Mild HCW | ns | 0.7163 |
| | Control vs. Severe/Critical | ns | 0.7528 |
| | Control vs. Negative inpatient | ns | >0.9999 |
| | Asymptomatic vs. Mild HCW | ns | 0.1164 |
| | Asymptomatic vs. Severe/Critical | ns | 0.3007 |
| | Asymptomatic vs. Negative inpatient | ns | 0.5417 |
| | Mild HCW vs. Severe/Critical | ns | >0.9999 |
| | Mild HCW vs. Negative inpatient | ns | >0.9999 |
| | Severe/Critical vs. Negative inpatient | ns | >0.9999 |

Kruskal Wallis test with Dunn's multiple comparison on magnitude of *ex vivo* interferon-gamma ELISpot responses

# Supplementary Table 4

| Dunn's multiple comparisons test | Summary of P value | Adjusted P Value |
|---|---|---|
| DMSO vs. S1 | **** | <0.0001 |
| DMSO vs. S2 | **** | <0.0001 |
| DMSO vs. M | **** | <0.0001 |
| DMSO vs. NP | **** | <0.0001 |
| DMSO vs. ORF 3 | **** | <0.0001 |
| DMSO vs. ORF 6 | ns | >0.9999 |
| DMSO vs. ORF 7 | ns | 0.0669 |
| DMSO vs. ORF 8 | ** | 0.0054 |
| S1 vs. S2 | ns | >0.9999 |
| S1 vs. M | ns | >0.9999 |
| S1 vs. NP | ns | >0.9999 |
| S1 vs. ORF 3 | *** | 0.001 |
| S1 vs. ORF 6 | **** | <0.0001 |
| S1 vs. ORF 7 | **** | <0.0001 |
| S1 vs. ORF 8 | **** | <0.0001 |
| S2 vs. M | ns | >0.9999 |
| S2 vs. NP | ns | >0.9999 |
| S2 vs. ORF 3 | ** | 0.0095 |
| S2 vs. ORF 6 | **** | <0.0001 |
| S2 vs. ORF 7 | **** | <0.0001 |
| S2 vs. ORF 8 | **** | <0.0001 |
| M vs. NP | ns | >0.9999 |
| M vs. ORF 3 | **** | <0.0001 |
| M vs. ORF 6 | **** | <0.0001 |
| M vs. ORF 7 | **** | <0.0001 |
| M vs. ORF 8 | **** | <0.0001 |
| NP vs. ORF 3 | *** | 0.0009 |
| NP vs. ORF 6 | **** | <0.0001 |
| NP vs. ORF 7 | **** | <0.0001 |
| NP vs. ORF 8 | **** | <0.0001 |
| ORF 3 vs. ORF 6 | *** | 0.0007 |
| ORF 3 vs. ORF 7 | ns | 0.1732 |
| ORF 3 vs. ORF 8 | ns | >0.9999 |
| ORF 6 vs. ORF 7 | ns | >0.9999 |
| ORF 6 vs. ORF 8 | ns | >0.9999 |
| ORF 7 vs. ORF 8 | ns | >0.9999 |

Kruskal Wallis test with Dunn's multiple comparison on frequency of proliferative CD4+ T cells

# Supplementary Table 5

| Dunn's multiple comparisons test | Summary of P value | Adjusted P Value |
|---|---|---|
| DMSO vs. S1 | **** | <0.0001 |
| DMSO vs. S2 | **** | <0.0001 |
| DMSO vs. M | **** | <0.0001 |
| DMSO vs. NP | **** | <0.0001 |
| DMSO vs. ORF 3 | **** | <0.0001 |
| DMSO vs. ORF 6 | ns | >0.9999 |
| DMSO vs. ORF 7 | ns | >0.9999 |
| DMSO vs. ORF 8 | ns | 0.7346 |
| S1 vs. S2 | ns | >0.9999 |
| S1 vs. M | ns | >0.9999 |
| S1 vs. NP | ns | >0.9999 |
| S1 vs. ORF 3 | * | 0.0291 |
| S1 vs. ORF 6 | **** | <0.0001 |
| S1 vs. ORF 7 | **** | <0.0001 |
| S1 vs. ORF 8 | **** | <0.0001 |
| S2 vs. M | ns | >0.9999 |
| S2 vs. NP | ns | >0.9999 |
| S2 vs. ORF 3 | ns | 0.0842 |
| S2 vs. ORF 6 | **** | <0.0001 |
| S2 vs. ORF 7 | **** | <0.0001 |
| S2 vs. ORF 8 | *** | 0.0001 |
| M vs. NP | ns | >0.9999 |
| M vs. ORF 3 | * | 0.0178 |
| M vs. ORF 6 | **** | <0.0001 |
| M vs. ORF 7 | **** | <0.0001 |
| M vs. ORF 8 | **** | <0.0001 |
| NP vs. ORF 3 | ** | 0.0018 |
| NP vs. ORF 6 | **** | <0.0001 |
| NP vs. ORF 7 | **** | <0.0001 |
| NP vs. ORF 8 | **** | <0.0001 |
| ORF 3 vs. ORF 6 | *** | 0.0004 |
| ORF 3 vs. ORF 7 | * | 0.0268 |
| ORF 3 vs. ORF 8 | ns | 0.3482 |
| ORF 6 vs. ORF 7 | ns | >0.9999 |
| ORF 6 vs. ORF 8 | ns | >0.9999 |
| ORF 7 vs. ORF 8 | ns | >0.9999 |

Kruskal Wallis test with Dunn's multiple comparison on frequency of proliferative CD8+ T cells

# Supplementary Table 6

| | | | CD4+ T cells | | | | | | | | CD8+ T cells | | | | | | | | | | | | |
|---|---|---|---|---|---|---|---|---|---|---|---|---|---|---|---|---|---|---|---|---|---|---|---|
| ID | Anti-spike_IgG | Total ELISpot | S1 | S2 | M | N | ORF 3 | ORF 6 | ORF 7 | ORF 8 | S1 | S2 | M | N | ORF 3 | ORF 6 | ORF 7 | ORF 8 | Symptoms | Sex | Age | symptoms to pcr test(days) | Days since symptom onset |
| 1† | 4 | 318 | 48.30 | 19.60 | 8.40 | 0.00 | 2.97 | 0.00 | 0.00 | 0.00 | 4.18 | 2.13 | 3.89 | 0.00 | 0.00 | 0.00 | 0.00 | 0.00 | myalgia, cough, night sweats | m | 40 | 6 | 107 |
| 2 | 1 | 1018 | 0.00 | 3.95 | 0.00 | 0.00 | 0.00 | 0.00 | 0.00 | 0.00 | 0.00 | 0.00 | 0.00 | 0.00 | 0.00 | 0.00 | 0.00 | 0.00 | cough, fatigue | m | 23 | 61 | 111 |
| 3 | 1 | 55 | 0.00 | 15.18 | 4.84 | 6.90 | 0.00 | 0.00 | 0.00 | 0.00 | 0.00 | 1.45 | 1.16 | 1.36 | 0.00 | 0.00 | 0.00 | 0.00 | sore throat, cough | m | 33 | 56 | 118 |
| 4† | 1 | 35 | 2.37 | 6.85 | 0.00 | 17.28 | 0.00 | 0.00 | 1.71 | 0.00 | 1.07 | 1.69 | 0.00 | 1.79 | 0.00 | 0.00 | 1.22 | 0.00 | fatigue, fever, shortness of breath | f | 29 | 2 | 83 |
| 5† | 1 | 1208 | 10.15 | 37.55 | 24.45 | 1.84 | 0.00 | 0.00 | 6.53 | 0.00 | 1.20 | 3.17 | 2.14 | 0.00 | 0.00 | 0.00 | 1.23 | 0.00 | sore throat, fatigue, headache | f | 24 | 1 | 82 |
| 6† | 1 | 90 | 30.49 | 15.59 | 13.09 | 7.19 | 2.48 | 17.29 | 0.00 | 25.79 | 1.08 | 0.00 | 0.00 | 0.00 | 0.00 | 0.00 | 0.00 | 0.00 | night sweats, fever, sore throat, anorexia, myalgia | f | 34 | 55 | 120 |
| 7 | 1 | 8 | Not Tested | | | | | | | | Not Tested | | | | | | | | fever, cough, sore throat, malaise, altered sense of taste | m | 33 | 36 | 126 |
| 8 | 1 | 105 | 0.00 | 15.08 | 1.19 | 0.00 | 2.13 | 1.82 | 4.10 | 0.00 | 0.00 | 1.71 | 0.00 | 0.00 | 1.11 | 0.00 | 0.00 | 0.00 | cough, fever | m | 34 | 128 | 140 |
| 9 | 1 | 90 | 0.00 | 0.00 | 1.31 | 0.00 | 1.77 | 0.00 | 0.00 | 1.46 | 0.00 | 0.00 | 0.00 | 0.00 | 0.00 | 0.00 | 0.00 | 0.00 | fever, chills, cough | m | 29 | 36 | 134 |
| 10 | 1 | 67.5 | Not Tested | | | | | | | | Not Tested | | | | | | | | fever, lethergy, dizziness, fatigue | m | 25 | 51 | 143 |

† PBMC samples from these volunteers were analysed from cryopreserved samples.

Clinical information and ELISpot and proliferation assay responses for highly exposed doctors used in this study

# Supplementary Table 7

| | S | M | NP |
|---|---|---|---|
| **OC43** | AOL02453 | AAT84365 | AGT51654 |
| **NL63** | AIW52836 | ALJ53434 | AAS58181 |
| **229E** | AIW52754 | AFR79253 | AFR79254 |
| **HKU1** | ABD75513 | ABD75500 | AGT17773 |

Accession ID for consensus sequence for seasonal coronaviruses

**Other members of the Oxford Immunology Network Covid-19 Response T cell Consortium**

Nicholas P. Day[1], Wentao Chen[2], Jian Luo[2], Helen McShane[3], Richard J. Cornall[4], Rachel Etherington[4], Hasham Koohy[4], Prathiba Kurupati[4], Chloe Hyun-Jung Lee[4], Giorgio Napolitani[4], Yanchun Peng[4], Mariana Pereira Pinho[4], Emmanouela Repapi[4], Timothy Rostron[4], Mariolina Salio[4], Paul Sopp[4], Zixi Yin[4], Maria Aggelakopoulou[5], Persephone Borrow[5], Danning Dong[5], Geraldine Gillespie[5], Guihai Liu[5], Chang Liu[5], Cesar Lopez-Camacho[5], Andrew McMichael[5], Wayne Paes[5], Guido Paeson[5], Isabela Pedroza-Pacheco[5], Luzheng Xue[5], Hongbing Yang[5], Xuan Yao[5], Matthew Edmans[6], Peter Simmonds[6], Yi-Ling Chen[7], Ushani Rajapaksa[7], Dannielle Wellington[7], Benedikt Kessler[8], Teresa Lockett[9], Wanwisa Dejnirattisai[10], Jonathan Grimes[10], Julian C. Knight[10], Juthathip Mongkolsapaya[10], Jose Slon-campos[10], Dave Stuart[10], Piyada Supasa[10], Ping Zhang[10], Yuguang Zhao[10], Robert Levin[11]

**Affiliation**

1. Centre for Tropical Medicine and Global Health, Nuffield Dept. of Clinical Medicine, University of Oxford, UK
2. Experimental Medicine, Nuffield Department of Clinical Medicine, University of Oxford, UK
3. Jenner Institute, University of Oxford, UK
4. MRC Human Immunology Unit, MRC Weatherall Institute of Molecular Medicine, University of Oxford, UK
5. Nuffield Department of Clinical Medicine, University of Oxford, UK
6. Peter Medawar Building for Pathogen Research, Nuffield Dept. of Clinical Medicine, University of Oxford, UK
7. Radcliff Department of Medicine, University of Oxford, UK
8. Target Discovery Institute, University of Oxford, UK
9. Thames Valley and South Midlands Clinical Research Network
10. Wellcome Centre for Human Genetics, University of Oxford, UK
11. Worthing Hospital, Worthing, UK
