# Supplementary material for: T cell assays differentiate clinical and subclinical SARS-CoV-2 infections from cross-reactive antiviral responses
Source: Nat Commun. 2021 Apr 6;12:2055. doi: 10.1038/s41467-021-21856-3 (PMC8024333; doi:10.1038/s41467-021-21856-3)
Supplement: Supplementary file 3 — Reporting Summary [file 41467_2021_21856_MOESM3_ESM.pdf]

nature research

Corresponding author(s): Paul.Klenerman@ndm.ox.ac.uk

Last updated by author(s): Jan 27, 2021

Reporting Summary

Nature Research wishes to improve the reproducibility of the work that we publish. This form provides structure for consistency and transparency in reporting. For further information on Nature Research policies, see our Editorial Policies and the Editorial Policy Checklist.

Statistics

For all statistical analyses, confirm that the following items are present in the figure legend, table legend, main text, or Methods section.

n/a	Confirmed	
☐	☒	The exact sample size (*n*) for each experimental group/condition, given as a discrete number and unit of measurement
☐	☒	A statement on whether measurements were taken from distinct samples or whether the same sample was measured repeatedly
☐	☒	The statistical test(s) used AND whether they are one- or two-sided<br>*Only common tests should be described solely by name; describe more complex techniques in the Methods section.*
☒	☐	A description of all covariates tested
☐	☒	A description of any assumptions or corrections, such as tests of normality and adjustment for multiple comparisons
☐	☒	A full description of the statistical parameters including central tendency (e.g. means) or other basic estimates (e.g. regression coefficient) AND variation (e.g. standard deviation) or associated estimates of uncertainty (e.g. confidence intervals)
☐	☒	For null hypothesis testing, the test statistic (e.g. *F*, *t*, *r*) with confidence intervals, effect sizes, degrees of freedom and *P* value noted<br>*Give P values as exact values whenever suitable.*
☒	☐	For Bayesian analysis, information on the choice of priors and Markov chain Monte Carlo settings
☒	☐	For hierarchical and complex designs, identification of the appropriate level for tests and full reporting of outcomes
☒	☐	Estimates of effect sizes (e.g. Cohen's *d*, Pearson's *r*), indicating how they were calculated

Our web collection on statistics for biologists contains articles on many of the points above.

Software and code

Policy information about availability of computer code

Data collection	ELISPOT - AID ELISpot Reader (v.4.0); Flow Cytometry - BD LSR II/DIVA v6, Miltenyi MACSQuant analyser 10
Data analysis	Flow Cytometry - Flowjo v10.7.1, Pestel v2.0 and Spice v6.0 Prism v8.4.3, Matlab version R2018b, Gen5 v3.09

For manuscripts utilizing custom algorithms or software that are central to the research but not yet described in published literature, software must be made available to editors and reviewers. We strongly encourage code deposition in a community repository (e.g. GitHub). See the Nature Research guidelines for submitting code & software for further information.

Data

Policy information about availability of data

All manuscripts must include a data availability statement. This statement should provide the following information, where applicable:

- Accession codes, unique identifiers, or web links for publicly available datasets
- A list of figures that have associated raw data
- A description of any restrictions on data availability

The source data from all the main figures and supplementary figures will be made available with the publication.
SARS-CoV-2 sequence was downloaded from GenBank (accession number: NC_045512 https://www.ncbi.nlm.nih.gov/gene/?term=NC_045512).

Field-specific reporting

Life sciences study design

All studies must disclose on these points even when the disclosure is negative.

Sample size	We designed a study aimed at elucidating SARS-COV-2 immune response in PCR+ frontline healthcare workers (HCWs) as such no sample size calculation was performed. All HCWs who had tested PCR+ to SARS-COV-2 and were consented to participate in the study were recruited as feasibility allowed. In total we report 168 SARS-CoV-2 PCR positive subjects and 118 SARS-CoV-2 negative patients
Data exclusions	All subjects who were recruited were included. No exclusion criteria were established prior to testing. Subjects were only excluded on a per assay basis where sample availability limited testing as in the proliferation responses in the SARS-CoV-2 PCR+ volunteers and the exposed seronegative Health Care Workers (HCWs). In ELISPOT assays, samples with negative wells with >50sfu/million cells were excluded. ICS was performed on all donors that had an ELISpot responses to any SARS-CoV-2 peptide pool (Figure 4) or all PCR+ donors (Suplemetary figure 5). For proliferation assay, non-responders (negative values after background subtraction) were excluded from further analysis.
Replication	Standardised ELISpots assays were run in duplicates. ELISpot findings for uninfected subjects (negative controls) were reproduced independently in two other laboratories in the UK (Figure 5). Proliferation assays were successfully validated on 12 HCV seronegative subjects using 2 HCV-specific peptide pools (HCV NS3 and Core protein) to determine level of background noise from the assay. To acquire enough events, proliferation assays were set up as duplicates and then pooled for fluorescence staining and analysis. Each datapoint represents an individual donor and all experiments using biological replicates were successful. All antibodies used were of the same clone throughout the duration of the study and different vials (if from different lots) titrated to obtain similar frequencies and MFI. DMSO control with matching percent DMSO was also used in all assays to account for DMSO content in peptide pools.
Randomization	We performed no randomization. Samples were selected based on exposure (or lack of) and/or PCR+ to SARS-COV-2. Randomisation was not appropriate for this study. Our study subjects represent the general population in a real-world observational cohort without intervention.
Blinding	Blinding was not possible or relevant for this study looking at how T cell assays differentiate clinical and subclinical SARS-CoV-2 infections from cross-reactive antiviral responses. All SARS-COV-2 PCR+ samples came from the hospital, and so it was not possible to be blinded to SARS-COV-2 PCR status of the subjects

Reporting for specific materials, systems and methods

We require information from authors about some types of materials, experimental systems and methods used in many studies. Here, indicate whether each material, system or method listed is relevant to your study. If you are not sure if a list item applies to your research, read the appropriate section before selecting a response.

Materials & experimental systems

n/a	Involved in the study	
☐	☒	Antibodies
☒	☐	Eukaryotic cell lines
☒	☐	Palaeontology and archaeology
☒	☐	Animals and other organisms
☐	☒	Human research participants
☒	☐	Clinical data
☒	☐	Dual use research of concern

Methods

n/a	Involved in the study	
☒	☐	ChIP-seq
☐	☒	Flow cytometry
☒	☐	MRI-based neuroimaging

Antibodies

Antibodies used	CD3 FITC (Dilution: 1 in 50ul, Company: Biolegend, Catalogue number: 300440, Clone: UCHT1, Lot number: B227643), CD4 AF700 (Dilution: 0.5 in 50ul, Company: BD Bioscience, Catalogue number: 557922, Clone: RPA-T4, Lot number: B291544), CD8 PECY7 (Dilution 0.25 in 50ul, Company: Biolegend, Catalogue number: 301012, Clone: RPA-T8, Lot number: 9009966), CD3 APC Fire 750 (Dilution: 0.15 in 50uL, Company: Biolegend, Catalogue number: 344840, Clone: SK7, Lot: B229306), CD4 PE Dazzle 594 (Dilution: 0.3 in 50uL, Company: Biolegend, Catalogue number: 300548, Clone: RPA-T4, Lot: B284876), CD8 PerCpCy5.5 (Dilution: 0.15 in 50uL, Company: Biolegend, Catalogue number: 301032, Clone: RPA-T8, Lot: B225562), CD107a BV421 (Dilution 0.16 in 200uL, Company: BD Biosciences, Catalogue number: 562623, Clone: H4A3, Lot: 0003034) , CD154 PECy7 (Dilution: 0.15 in 50uL, Company: Biolegend, Catalogue number: 310832, Clone: 24-31, Lot: B237827), IFN- APC (Dilution: 0.3 in 50uL, Company: BD Biosciences, Catalogue number: 554702, Clone: B27, Lot: 31187), IL-2 PE (Dilution: 2.5 in 50uL, Company: Biolegend, Catalogue number: 500307, Clone: MQ1-17H12, Lot: B272813), TNF FITC (Dilution: 0.08 in 50uL, Company: BD Biosciences, Catalogue number: 554512, Clone: Mab11, Lot: 8323911). Streptavidin alkaline phosphatase (Dilution: 1 in 1000ul, Company: Vector Labs, Catalogue number: SP-3020,Lot: ZG0309), rabbit anti-human whole IgG conjugated to alkaline phosphatase (Dilution: 1 in 5000ul, Company: Sigma, Catalogue number A3187-1ml, Lot number: SLCG3198)
Validation	Use of these antibodies on human subjects are abundant in published literature and the manufacturer websites. All antibodies are further titrated upon receipt to validate performance<br>CD3 FITC https://www.biolegend.com/en-us/products/fitc-anti-human-cd3-antibody-863?GroupID=GROUP28<br>CD4 AF700 https://www.bdbiosciences.com/eu/applications/research/t-cell-immunology/th-1-cells/surface-markers/human/alexa-fluor-700-mouse-anti-human-cd4-rpa-t4/p/557922

CD8 PECY7 https://www.biolegend.com/en-us/products/pe-cyanine7-anti-human-cd8a-antibody-838
Live/Dead Aqua https://www.thermofisher.com/order/catalog/product/L34957#/L34957
CD3 APC Fire 750 https://www.biolegend.com/en-gb/search-results/apc-fire-750-anti-human-cd3-antibody-13004
CD4 PE Dazzle 594 https://www.biolegend.com/en-us/products/pe-dazzle-594-anti-human-cd4-antibody-9780?GroupID=GROUP28
CD8 PerCpCy5.5 https://www.biolegend.com/en-us/products/percp-cyanine5-5-anti-human-cd8a-antibody-4222?GroupID=GROUP28
CD107a BV421 https://www.bdbiosciences.com/us/applications/research/intracellular-flow/intracellular-antibodies-and-isotype-controls/anti-human-antibodies/bv421-mouse-anti-human-cd107a-h4a3/p/562623
CD154 PECy7 https://www.biolegend.com/en-us/products/pe-cyanine7-anti-human-cd154-antibody-9233
IFN- https://www.bdbiosciences.com/us/applications/research/t-cell-immunology/th-1-cells/intracellular-markers/cytokines-and-chemokines/human/apc-mouse-anti-human-ifn--b27/p/554702
IL-2 PE https://www.biolegend.com/it-it/products/pe-anti-human-il-2-antibody-1351
TNF FITC https://www.bdbiosciences.com/us/applications/research/t-cell-immunology/th-1-cells/intracellular-markers/cytokines-and-chemokines/human/fitc-mouse-anti-human-tnf-mab11/p/554512

Human research participants

Policy information about studies involving human research participants

Population characteristics

We recruited donors who were exposed to SARS-COV-2 (PCR+/-) and unexposed seronegative control during and pre COVID19 pandemic. All population characteristics are described in supplementary table S1 and S7

Recruitment

Healthcare workers at Oxford University Hospitals NHS Foundation Trust who tested positive for SARS-CoV-2 following either presentation to the hospital's Occupational Health Department with symptoms or had a positive PCR test on the staff screening programme were asked to indicate whether they were willing to be contacted by researchers. Individuals who agreed to be contacted received an email invitation to participate in the study. Subjects recruited from the staff screening programme were classified as asymptomatic if they did not report any symptoms of COVID-19 (including fever, shortness of breath, cough, loss of taste or smell, sore throat, coryza or diarrhoea), either prior to staff screening or in the seven days following testing positive. In total 126 symptomatic and 33 asymptomatic subjects were recruited for this study. In addition, 9 hospitalised patients with WHO severe or critical COVID-19 were studied.

30 healthy control subjects in Oxford and 13 in Sheffield with no history of COVID-19 symptoms and no antibodies to SARS-CoV-2 spike protein detected by IgG ELISA were recruited. In addition, archived samples from 12 healthy control subjects in Oxford who donated blood in the pre-pandemic period (2008-2019) were studied, alongside 48 healthy control subjects from the pre-pandemic period in Liverpool. Hospital in-patients with PCR-confirmed SARS-CoV-2 infection or PCR-negative inpatient control subjects were recruited by the study team.

10 acute medicine doctors, who worked in patient facing services during the pandemic and experienced symptoms compatible with COVID-19, but did not receive PCR testing at the time of symptoms or tested negative, and were anti-spike IgG negative two months after the pandemic peak, were recruited as highly exposed seronegative participants

Potential bias that could impact results may include the age and gender of the SARS-CoV-2+ volunteers. However, our analysis of the ELISPOT data show that in our cohort, there was no statistically significant difference in anti-SARS-CoV-2 responses when the data was analysed based on age and gender (Supp. Fig 1g and h).

Ethics oversight

Human study protocols were approved by the research ethics committee (REC) at Yorkshire & The Humber - Sheffield (GI Biobank Study 16/YH/0247). The study was conducted according to the principles of the Declaration of Helsinki (2008) and the International Conference on Harmonization (ICH) Good Clinical Practice (GCP) guidelines. Written informed consent was obtained for all patients enrolled in the study.

Note that full information on the approval of the study protocol must also be provided in the manuscript.

Flow Cytometry

Plots

Confirm that:

- [x] The axis labels state the marker and fluorochrome used (e.g. CD4-FITC).
- [x] The axis scales are clearly visible. Include numbers along axes only for bottom left plot of group (a 'group' is an analysis of identical markers).
- [x] All plots are contour plots with outliers or pseudocolor plots.
- [x] A numerical value for number of cells or percentage (with statistics) is provided.

Methodology

Sample preparation

Peripheral blood mononuclear cells were isolated by density gradient centrifugation. Plasma was collected and spun at 2000g for 10 minutes to remove platelets before freezing at -80°C for later use. following PBMC isolation, cells were resuspended in

	R10 and counted using the Guava® ViaCountTM assay on the Muse Cell Analyzer (Luminex Cooperation). For functional assays, PBMC were stimulated with three groups of peptide pool for SARS-CoV-2: (1) Spike: 15-mers overlapping by 10 amino acid residues for spike (S), divided into 12 "minipools" P1-P12 (Proimmune), and grouped into pools S1 (P1-6) and S2 (P7-12) for some assays (2) Structural and accessory proteins: 12-20-mer peptides overlapping by 10 amino acid residues for membrane protein (M), nucleoprotein (NP), envelope (E) protein, open reading frame (ORF) 3, 6, 7 and 8 (Proimmune) and (3) Predicted epitope pools: predicted CD4+ and CD8+ pools from the Sette laboratory, La Jolla Institute, CA, all used at a final concentration of 1-2ug/ml per peptide depending on the assay. Lyophilised peptides were reconstituted in DMSO (Sigma)
Instrument	BD LSR II and Miltenyi MACQuant
Software	FlowJo version 10.7.1
Cell population abundance	No manipulation of samples post PBMC isolation was performed. All assays were set up with fresh or cryopreserved total PBMC.
Gating strategy	For proliferation assay - Lymphocyte gating was done using FSC and SSC. Doublets were then gated out using FSC gates. This was followed by a live lymphocyte gate using CD3 and live/dead cell dyes Following this, separation onto CD4 and CD8 lineages and subsequent analysis was performed This is represented in supplementary figure 2. For ICS assay, representative gates are available in supplementary figure

☒ Tick this box to confirm that a figure exemplifying the gating strategy is provided in the Supplementary Information.
